# Assessing the Legal Aspects of Information Security Requirements for Health Care in 3 Countries: Scoping Review and Framework Development

**DOI:** 10.2196/30050

**Published:** 2022-05-25

**Authors:** Prosper Kandabongee Yeng, Muhammad Ali Fauzi, Luyi Sun, Bian Yang

**Affiliations:** 1 Department of Information Security and Communication Technology Norwegian University of Science and Technology Gjovik Norway

**Keywords:** legal requirement, information security, healthcare, security practice

## Abstract

**Background:**

The loss of human lives from cyberattacks in health care is no longer a probabilistic quantification but a reality that has begun. In addition, the threat scope is also expanding to involve a threat of national security, among others, resulting in surging data breaches within the health care sector. For that matter, there have been provisions of various legislation, regulations, and information security governance tools such as policies, standards, and directives toward enhancing health care information security–conscious care behavior among users. Meanwhile, in a research scenario, there are no comprehensive required security practices to serve as a yardstick in assessing security practices in health care. Moreover, an analysis of the holistic view of the requirements that need more concentration of management, end users, or both has not been comprehensively developed. Thus, there is a possibility that security practice research will leave out vital requirements.

**Objective:**

The objective of this study was to systematically identify, assess, and analyze the state-of-the-art information security requirements in health care. These requirements can be used to develop a framework to serve as a yardstick for measuring the future real security practices of health care staff.

**Methods:**

A scoping review was, as a result, adopted to identify, assess, and analyze the information security requirement sources within health care in Norway, Indonesia, and Ghana.

**Results:**

Of 188 security and privacy requirement sources that were initially identified, 130 (69.1%) were fully read by the authors. Subsequently, of these 188 requirement documents, 82 (43.6%) fully met the inclusion criteria and were accessed and analyzed. In total, 253 security and privacy requirements were identified in this work. The findings were then used to develop a framework to serve as a benchmark for modeling and analyzing health care security practices.

**Conclusions:**

On the basis of these findings, a framework for modeling, analyzing, and developing effective security countermeasures, including incentivization measures, was developed. Following this framework, research results of health care security practices would be more reliable and effective than relying on incomprehensive security requirements.

## Introduction

### Background

There have been enormous gains in the application of information technology (IT) in health care in various areas such as decision support, telemedicine, electronic health record (EHR) management, chronic disease management with medical devices, drugs, and vaccine production [1-3]. However, cyberattacks in health care and their related adverse impact are a significant problem, especially in the midst of the infamous COVID-19 pandemic [4]. For example, Brno University Hospital in the Czech Republic was recently attacked, and cyberattackers were believed to have used spear phishing to gain access and deployed ransomware, which encrypted the data in the entire hospital network [5]. The hospital was compelled to shut down and battle with the cyberattack to restore its data. Even though the hospital was one of the COVID-19 treatment centers, the incident apparently prevented them from providing health care services during the attack period. Following that, there were other cyberattacks on the World Health Organization, Hammersmith Medicines Research Group in the United Kingdom (a COVID-19 vaccine trial group), the US Health and Human Services Department, Paris Hospital Authority in France, Bam Construct and Interserve (a COVID-19 hospital construction company), and Babylon Health (a hospital appointment and teleconsultation videoconferencing system) in the United Kingdom [6].

In addition, cybersecurity and privacy issues in health care have become a global concern as data breaches in health care continue to surge. In 2017, approximately 5 million health care records were compromised globally [1-3]. This tripled in 2018 to approximately 15 million, and the number of compromised health care records continues to increase yearly [3]. In addition, the cost associated with data breaches (eg, cost of detection of breaches, cost of fines paid in data breaches, cost of recovery, and payment of ransoms) is the highest in health care among various industries [7].

Data breaches and security issues in health care have major consequences on confidentiality, integrity, and availability (CIA). This usually perturbs the data subjects, the health care organizations, and the laws of the countries involved [8,9]. The adverse impact on data subjects includes situations in which the stolen data can be used as a means of pressure to demand other goals by criminals. Recently, an instance occurred in Finland [10], where stolen medical records were used by cybercriminals to pressure the data owners for money. Unauthorized persons can also disrupt the proper functioning of health care operations, such that the net effect can result in the loss of a patient’s life. A related instance occurred in Germany, where a hospital’s IT systems were hit by ransomware, which resulted in the death of a patient due to the unavailability of the health care system at the time of need [11]. Mutual trust and confidentiality between health care providers and patients [12-15], economic losses [10,15,16], privacy issues [9,17], and unreliable medical records [11,18,19] or medical devices [3] are some of the effects often faced by data subjects during cyberattacks in health care. It could be much disheartening for patients to battle against their medical conditions, and at the same time, they have to battle with their privacy issues arriving from cyberattacks. Mutual trust with data between health care professionals and patients is very cardinal in terms of good-quality health provision. Health care professionals depend on the accuracy and comprehensiveness of the information provided by patients for therapeutic measures [13]. Therefore, health care providers are required to store large quantities of sensitive personal information of patients [14]. Similarly, patients trust that their personal information disclosed for medical reasons is to be kept confidential [15]. Sadly, this mutual trust in relation to patients’ data is often broken in data breaches [15,16].

Furthermore, health care systems are targeted for various computer crimes with the intention of stealing, altering, hindering, and disrupting data or other functions [5,11]. The consequences of cyberattack on health care organizations include loss of trust, credibility, and confidence from stakeholders; in addition, the financial impact on their organization and the hospital may face regulatory sanctions [9,20,21] if due care and due process were not followed. Health care issues emanating from cyberattacks can also undermine a nation’s health care policy as a whole, as the unavailability of health care systems could undermine the rights of citizens to health care [14,22].

In addition, laws have been enacted in various jurisdictions to protect the privacy of people in their countries [18,20,23]. However, data breaches in health care disrupt all these measures. According to the forecast of the International Organization for Standardization (ISO), the estimated annual losses from cybercrime could soon reach USD2 trillion [14] with countless daily breaches [19]. This forecast is in resonance with the current trend of the cost of data breaches of which health care is in the lead [7].

In this light, the European Union (EU) classified health care as an essential service having foreseen cyberattack on health care as a threat to national security [22]. This requires member states and the European Economic Area–affiliated member states to develop a culture of security across services that are vital for the economy and society and rely heavily on information and communication technology (ICT).

To maintain security in health care, various laws exist, including regulations, directives, statutory and constitutional laws, and various information security governance measures such as policies, standards, guidelines, and best practices, called “information security requirement” in this study. These were developed to prevent information security issues in health care. Owing to various cybersecurity issues, various efforts have been made to measure the security practices of health care staff [3,16,24-29], as they are the weakest link in the security chain [30,31]. However, these activities require a benchmark in the context of legal requirements in information security in health care that can be used as the measuring standard in such studies. For example, to create a questionnaire to measure health care staff’s cybersecurity practices, the content of the questionnaire could be derived from the legal requirements. Therefore, the question is, what is the benchmark that is to be used as a yardstick for measuring the security compliance level of health care staff and to what extent have these security requirements been incorporated at the organizational level where these security requirements are to be followed?

Security violations in health care facilities are not due to a lack of rule-based requirements but due to a lack of compliance with rules and in some cases due to technical vulnerabilities that could not be addressed by law, requiring an investigation as to why the challenges exist in complying with these rules. In measuring the cybersecurity practices of the health care staff, a comprehensive security requirement is required. However, a noncomprehensive security requirement is sometimes relied on, which does not serve as an effective baseline. For instance, in a recent assessment of the security practice of health care in Norway [32], the study relied on the Health Register Act, the Health Personnel Act, the Patient Records Act, and the General Data Protection Regulation (GDPR). The study relied on some legal sources; however, other vital legal sources such as the Personal Data Act of Norway, the Network and Information Security Directive of EU, and the Medical Device Directive of EU, were not considered. Other related studies [33,34] have considered a legal requirement in their work, but no study has comprehensively and systematically conducted a study on legal requirements that can serve as a benchmark for assessing health care staff security practices.

The general objective of this study is therefore to address this gap by comprehensively identifying the required security requirements in health care through state-of-the-art studies to provide input for the development of a framework for analyzing health care security practice in the context of legal requirements. The remaining sections include background studies and a specification of the scope, contribution, and research questions. This is followed by the research methods, findings, and discussion of results. A framework for analyzing health care security practice in the context of legal requirements is then presented for real studies in the future.

The health care information of persons is one of the most sensitive personal information and therefore has special protection from various laws [14,23,35,36]. Laws are rules elected to be followed by members of a society to meet the needs of society while balancing individual rights to their self-determination [37]. Laws frown against certain behaviors and are enforced by a state or the governing body. Therefore, all categories of health care information system users are legally bound to comply with legal requirements of which a contrary act will attract the application of punitive measures [20,36,38]. Therefore, it is extremely important to consider legal requirements as the baseline in measuring the security practices of health care staff.

Owing to the numerous threats of attack in health care [1-6], there have been initiatives to measure the security practices of health care staff [16]. This is to help identify the security requirements that are not being complied with and further determine the challenges or reasons why these security measures are not being complied with. The results of this study will help in finding effective solutions to enhance the conscious care behavior of users. Security practice in this study refers to how users respond to or comply with security measures that have been established to meet the CIA requirement of systems and resources [16,24,26].

In assessing the security practices in health care, it is important to establish the scope of the hospital’s legal and ethical obligations in relation to information security and privacy management [16,24,37]. This requires a catalog of comprehensive security requirements to understand the state-of-the-art legal requirements, including regulations, directives, policies, and guidelines for the fortification of users in health care IT systems against cyberattacks.

A comprehensive state-of-the-art security requirement is needed [39,40]; otherwise, what will be the benchmark in assessing the security practice level of hospital users? Moreover, if there is a security breach in health care by a user based on a lack of knowledge of a security requirement, the organization can still be liable or legally responsible [41]. This means that the health care organization will continue to make restitution for related harm caused in the breach [41]. This calls for due care and due diligence [42,43] on the part of health care organizations. Due care is measures taken by an organization to ensure that all employees are aware of acceptable and nonacceptable security practices, whereas due diligence is reasonable measures that are taken by the organizations or people to meet the established security requirements imposed by law [37]. Health care organizations increase their risk of being liable if they fail to adopt due care and due diligence measures. This is necessary because health care tends to rely more on IT and the internet for efficiency; a larger number of people can be adversely affected in a security breach situation as internet-based solutions are globally reached, which therefore require security due diligence and due care [37,42,43].

### Type of Laws

Laws can be categorized based on their origins, such as constitutional law, statutory law, regulatory or administrative law, and common law, which is otherwise known as case law or precedents [37,44,45]. Constitutional law originates from the constitution of a state, bylaws, or a charter, but laws that originate from the legislative arm of governance with the mandate to make and publish laws of the country are known as statutory laws [37,44]. Furthermore, regulatory or administrative laws are created from the executive arm of the government or an authorized regulatory agency backed with executive orders and regulations [37,44]. Laws made from the judicial branch and boards based on the interpretation of law through the previous ruling of a higher court or boards are referred to as common law, case law, or precedents.

Statutory law can be further categorized into civil law and criminal law based on their association with individuals, groups, and the state [46]. Civil law has to do with issues between and among individuals and organizations [37,44] and includes contract law, employment law, and tort law. Tort law enables individuals to settle their issues in court on personal, physical, or financial matters. In such matters, restitution is settled in civil courts without the state’s involvement. At the same time, criminal law is enforced and prosecuted by the state and deals with violations that are harmful to society. In criminal law, the state acts on behalf of the plaintiff to obtain retribution for the plaintiff. For instance, in some jurisdictions, health care professionals are punished for criminal behavior if they disclose their clients’ information without good causes [47].

### Security Policies, Standards, Guidelines, Procedures, and Practices

In controlling information security in a health care organization, information security governance is usually adopted by organizations that use policies, standards, guidelines, procedures, and practices [37]. In various health care units, organizational policies function as the laws. Therefore, information security policies are required to be made and implemented to ensure that they are complete and appropriate and should be able to fairly apply to everyone in the workplace [37]. As laws, organizational policies must be completed with retributions, judicial practices, and sanctions to require compliance.

However, the variance between law and policy is that although ignorance of state law is not an excuse, ignorance of an organizational policy is an acceptable defense [37]. Therefore, to have an enforceable policy in an organization, the policy must be disseminated, reviewed, comprehended, complied with, and uniformly enforceable to all staff in the organization.

Information security policy directs how issues should be addressed and how IT resources should be used, but it does not define the proper operation or functioning of the system. How a software program should function is specified in the standard procedures and practices of the users’ manuals and systems documentation.

Policies specify acceptable and unacceptable information security practices at the organizational level and outline rules with the aim of protecting the organization’s information assets [48,49]. There are 3 types of information security policies [37,48,49]: the enterprise or organizational information security policy (EISP), issue-specific security policy (ISSP), and system-specific policy.

EISP is a general information security policy that contains the overall strategic direction, scope, and goal of the organizational information needs at a high level. In addition, EISP defines the legal requirements, outlines the responsibilities of the system administration of information security policy maintenance and practices, and outlines the responsibilities of the users.

While EISP is aimed toward addressing a broad scope of the entire organization’s security issues, ISSP provides detailed guidelines pertaining to the use of specific resources, such as processor or technology, for all members or users to comply with [37,48,49]. Some of these instances include email use, internet use, security measures against viruses, bringing your own devices, use of cloud computing, home use of company-owned devices, data retention policy, and media disposal policy.

EISP and ISSP still provide information security rules at a more general level when focusing on specific systems in the organization, and they do not address security issues concerning specific systems. This gap has been filled by system-specific policy, which provides adequate information or direction in complying with the security of specific systems in the organization [37,48-50]. System-specific policy focuses on one system such as EHR systems. In this context, system-specific policy, for instance, can be used to define the access control policy of the EHR system. Therefore, system-specific policy varies from system to system and is defined by management.

All these types of policies are effectively implemented using tools such as standards, guidelines, procedures, and practices [37,48-50]. Specifics that enable employees to comply with a security policy are known as information security standards, whereas guidelines are recommendations or examples provided to help users comply with a security policy. Practices are also recommendations or examples that are adopted from a reputable organization to help in complying with a policy, whereas procedures are step-by-step instructions users are to follow to accomplish a particular task in fulfillment of the security policy.

### Scope, Contribution, and Research Questions

In assessing the information security practice of health care staff, there is a need to determine the state of security practice in the health care organization and compare it to a benchmark to determine the level of compliance with information security of the health care staff of that organization. Therefore, we opine that the legal aspect of the information security requirement is necessary to serve as the yardstick in measuring health care staff’s security practices. A major reason is that a violation of any legal requirement has a huge consequence on the offending individual or company, including heavy fines, imprisonment, and payments of restitution. Therefore, aiming to comply with the legal aspect of information security requirements by using it as a yardstick will lead to unconscious compliance with the laws of that jurisdiction.

Information security requirement does not only involve legal requirements but also includes ethical security considerations of information system users [37]. However, this study focuses on the legal requirements of information security in health care such as constitutional law, statutory law, regulations, case law, and chatters. Other legal sources considered in this study include information security policies and their supported instruments, such as information security standards, guidelines, and practices.

This study seeks to address issues of incomprehensiveness in considering the legal requirements for analyzing health care security practices in Norway, Ghana, and Indonesia. This has become necessary, as there have been initiatives to measure the security practices of health care staff in these countries in various projects [16]. The problem is that there is no comprehensive and state-of-the-art study of the legal requirements of information security that can serve as a baseline for assessing security practices in health care. A random and nonsystematic approach to adopting legal information security requirements in real studies could undermine the quality of the study if the baseline for the measurement is wrong. Therefore, we adopted a comprehensive, systematic scoping review approach to establish our baseline legal requirements for future imperial studies and further developed a framework to guide future related studies.

## Methods

### Overview

A scoping review was conducted to explore information security and privacy requirement in health care following the PRISMA (Preferred Reporting Items for Systematic Reviews and Meta-Analyses) statement [1].

Various types of systematic studies include systematic mapping, scoping, and systematic literature review [51-54]. Systematic mapping studies rely on general research questions aimed at determining research trends or state-of-the-art studies as opposed to a scoping method that is based on the categorization of the study into topics [51,52], whereas systematic literature review aims to accumulate data with more specific research focus and synthesis. Therefore, in this study, a systematic scoping study was adapted. This section describes the methods and designs that were used to review the literature and conduct this study.

### Search Strategy

**Figure 1:**
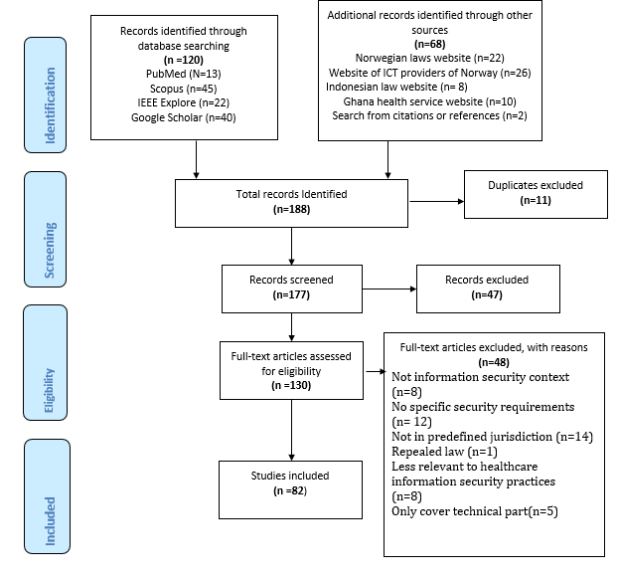
PRISMA (Preferred Reporting Items for Systematic Reviews and Meta-Analyses) diagram. ICT: information and communication technology.

The goal of the search is to search broadly to obtain comprehensive laws or rules termed here as *security requirements*. Therefore, we did not want to limit the identification of these requirements by searching through only scientifically published papers. This led to the inclusion of both scientific studies and other sources, shown in Figure 1. Therefore, the inclusion of scientific studies was intended to extract relevant laws. The sources of the security requirement were identified by conducting a literature search through several databases as follows: PubMed, Google Scholar, IEEE Xplore, and Scopus.

While reading the articles to identify the legal requirement, other relevant articles which were cited or referenced were also added in the studies and accounted for on the PRISMA diagram as *search from citations or references* as shown in Figure 1.

In addition, we also performed manual searching through several law databases by reading all the laws under the health care category and selecting the relevant ones. The databases used were as follows:

- Legal, regulations, and directive databases for EU and Norway [55]
- Legal, regulations, directive, policy, and code of conduct databases for hospitals in Norway [55]
- Legal, regulations, and directive databases for Indonesia [56]
- Legal, regulations, directive, policy, and code of conduct databases for hospitals in Indonesia [57]
- Legal, regulations, and directive databases for Ghana [58]
- Legal, regulations, directive, policy, and code of conduct databases for hospitals in Ghana [59,60]

The literature search was conducted without time restrictions. For searching the scientific paper databases, we used the following keywords in the search string: (*Information security* OR *Cyber security* OR *Computer security*) AND *Healthcare* AND *Information system* AND (*law* OR *Regulation* OR *Directive* OR *Policy* OR *Standard*) AND (*European Union* OR *Norway* OR *Indonesia* OR *Ghana*). Meanwhile, for searching through law databases, we did not use any keywords. Instead, we read all the laws under the health care category and selected the relevant ones. The literature search was conducted from December 2020 to February 2021.

### Eligibility Criteria

All studies involving laws, regulations, directives, policies, best practices, and standards in the health care security and privacy context in Norway and EU, Indonesia, or Ghana were eligible for review. The publication language was limited to English. Papers that did not meet the eligibility criteria or only described the technical part of security and privacy in health care without relying on legal or security governance requirements were excluded from the review. Only studies that describe the legal aspect of health care security and privacy in Norway and EU, Indonesia, or Ghana were eligible for review. Owing to the lack of resources, we focused on English scientific papers but only translated the identified local laws, which were relatively few.

### Study Selection and Data Extraction

A PRISMA flow diagram of the literature search process is shown in Figure 1. The titles and abstracts of articles from the databases were screened for eligibility. Then, all articles that passed the first screening entered full-text screening and data extraction. Data extraction was performed using a predesigned data collection form. For each qualified article, data on study characteristics, such as the first author and publication year, were extracted. Furthermore, we extracted information consisting of the article information, name and type of the legal document, legal document authority, security requirement, privacy requirement, health care user category, domain, responsibility level, security, and privacy requirement, which is referred to in this study as data categorization, as shown in Table 1.

**Table 1:** Data extraction field description.

No	Category	Description
1	Paper information	Name, authors, and publication year of the paper
2	Legal document name	The name of the legal documents found in the paper
3	Legal document type	This defines the category of law such as regulation, constitutional law, directive, statutory law, policy, and guidelines found in the paper
4	Legal document jurisdiction	The country in which the legal document applies
5	Security requirement	The requirement about information security found in the legal document
6	Privacy requirement	These are the measures or rules that seek to protect the dignity of patients. These include the right to consent and the right to be forgotten to preserve the privacy of an individual
7	Health care user category	The category of users with the primary responsibility to implement or comply with the related requirement. These include management, end users, and all users. The management category includes top management such as CEOs^a^, directors, managers, and officers with the responsibility of implementing and complying with the privacy and security requirement
8	Responsibility level	The user level is responsible for the requirement, and this defines the type of user category who is to take action to observe, enforce, implement, or comply with the security measure. Examples include management, end users, and all users. The management includes top-level staff such as the CEOs, directors, managers, and officers who are responsible for implementing and observing health care security practices. End users include all employees, consultants, suppliers, and others with access to the health system. All user-level categories include responsibilities that are concerned by management and end users
9	Security category	This refers to the security domain (eg, access control, security governance, access logs, and encryption) of the requirement
10	Privacy category	This refers to the privacy domain, such as consent and right to privacy, of the requirement and data protection

^a^CEO: chief executive officer.

### Data Categorization

Data categorization was developed based on the objective and thorough literature reviews and author discussions. The categories were defined exclusively to assess, analyze, and evaluate the study, as shown in Table 1.

### Literature Evaluation

After data extraction, all researchers independently checked the extracted data. A discussion between all researchers was held to resolve all discrepancies. The selected articles were assessed, analyzed, and evaluated based on the defined categories in Table 1 to evaluate the state-of-the-art security and privacy requirements. The percentages of the attributes of the categories were calculated based on the total number of counts (n) of each type of attribute. Some studies used multiple categories; therefore, the number of counts of these categories exceeded the total number of articles on the requirements presented in the study.

After data extraction, all researchers independently checked the extracted data. A discussion among all researchers was held to resolve any discrepancies.

## Results

### Study Selection

A total of 188 articles were identified through the literature search of the 10 databases. After duplicate deletion, 94.1% (177/188) of the articles remained for the next step. Titles and abstracts screening yielded in the exclusion of 26.6% (47/177) of the articles for not meeting eligibility criteria. Hence, 73.4% (130/177) of the articles entered the full-text screening for eligibility. After the second screening, 36.9% (48/130) of the articles were eliminated from the review for various reasons, with the main reasons being not in predefined jurisdictions (14/48, 29%) and not having specific information security and privacy requirements (12/48, 25%). To retrieve the list of excluded papers, a request can be sent to the authors. Finally, of the 130 articles in the full-text reading stage, 82 (63.1%) met the eligibility criteria and were included for review, as shown in Figure 1.

### Study Characteristics

Of the 82 articles, 36 (44%) were scientific studies and the others were legal documents. A total of 75 unique legal documents were identified, including case law (n=1, 1%), charter (n=1, 1%), code of conduct (n=1, 1%), directives (n=7, 9%), guidelines (n=4, 5%), policies (n=27, 36%), recommendation (n=1, 1%), regulations (n=13, 17%), standards (n=4, 5%), and statutory law (n=16, 21%), as shown in Multimedia Appendix 1 and Table 2. The distribution of law jurisdictions is depicted in Multimedia Appendix 2 and Table 3. Of the 75 legal documents, 35 (47%) are from Norway, 9 (12%) from Ghana, 11 (15%) from Indonesia, and 17 (23%) from the EU and 3 (4%) are international laws, as presented in Table 4, Table 5, Table 6, Table 7, and Table 8, respectively. In total, 253 requirements were extracted from the legal documents, consisting of 173 (68.4%) security requirements and 80 (31.6%) privacy requirements, as shown in Multimedia Appendix 3. As shown in Multimedia Appendix 4, of the 173 security requirements, 143 (82.7%) are the management’s responsibility to fulfill, 1 (0.6%) is the end users’ responsibility, and 29 (16.8%) are all users’ (management and end users) responsibility. Meanwhile, as shown in Multimedia Appendix 4, of the 80 privacy requirements, 70 (88%) need to be fulfilled by the management, 1 (1%) is the end users’ responsibility, and 9 (11%) are all users’ responsibility. Legal requirements are shown in Table 9; in addition, we classified the requirements into several categories, as shown in Tables 10 and 11.

**Table 2:** Types of laws (n=75).

No	Type of law	Count, n (%)
1	Case law	1 (1)
2	Charter	1 (1)
3	Code of conduct	1 (1)
4	Directive	7 (9)
5	Guideline	4 (5)
6	Policy	27 (36)
7	Recommendation	1 (1)
8	Regulation	13 (17)
9	Standard	4 (5)
10	Statutory law	16 (21)

**Table 3:** Count of laws based on jurisdiction (n=75).

No	Country	Count of laws, n (%)
1	Norway	35 (47)
2	Ghana	9 (12)
11	Indonesia	11 (15)
4	European Union	17 (23)
5	International	3 (4)

**Table 4:** Legal documents from Norway.

No	Legal document	Type
1	Code of conduct for information security and data protection in the health care and care services sector version 6.0 [61]	Code of conduct
2	Ministry of Government Administration, Reform and Church Affairs’ requirements specification for PKI^a^ for the public sector [62]	Guidelines
3	General principle to regional control system for information security and privacy [63]	Policy
4	Safety regulator legislation applicable to the enterprise group [63]	Policy
5	Organization of information security work [63]	Policy
6	Safety goals and level for acceptable risk of information security [63]	Policy
7	Security strategy [63]	Policy
8	Security instructions (signed version) [63]	Policy
9	ICT^b^ services and information security for medical devices [63]	Policy
10	Requirements specification—ICT services and information security for MTU^c^ [63]	Policy
11	Security principles and requirements for ICT infrastructure and applications [63]	Policy
12	Anonymization of health and personal information [63]	Policy
13	Use of data processor—treatment of personal information at other legal entity [63]	Policy
14	Use of email and fax [63]	Policy
15	Use of mobile phones [63]	Policy
16	Basis for posting in journal [63]	Policy
17	Storage, archiving, and deletion of health and personal information [63]	Policy
18	Crypto policy [63]	Policy
19	Password policy for the health trusts in Health South-East	Policy
20	Guidance for approval of data processing from secure third countries [63]	Policy
21	Requirements for coded research data	Policy
22	Use of email, fax, and SMS text messaging for communication with and about patients [63]	Policy
23	Regional policy for publishing and public services and DMZ^d^ [63]	Policy
24	Description of identification procedure in Health South-East [63]	Policy
25	Use of logs for administrative purposes	Policy
26	Internal control information security [63]	Policy
27	Logging of activity and control of logs [63]	Policy
28	Regional security policy for cloud services [63]	Policy
29	Regulations relating to the Processing of Personal Data [64]	Regulation
30	Norwegian Personal Health Data Filing System Act [16,65,66]	Statutory law
31	Act relating to Patients’ Rights	Statutory law
32	Act relating to the Processing of Personal Data [18]	Statutory law
33	Health Care Personnel Act [67,68]	Statutory law
34	Health Research Act [16]	Statutory law
35	Act relating to Public Supervision of the Health Service	Statutory law

^a^PKI: public key infrastructure.

^b^ICT: information and communication technology.

^c^MTU: medical technical equipment.

^d^DMZ: demilitarilized zone.

**Table 5:** Legal documents from Ghana.

No	Legal document	Type
1	The GHS^a^ Patient’s Charter	Charter
2	The Medical Profession Regulation and the Infectious Diseases, Cap 78	Regulation
3	The Ghana National Health Insurance Regulations of 2004	Regulation
4	Data Protection Act of Ghana 843	Statutory law
5	The Republic of Ghana’s Constitution	Statutory law
6	The National Identification Authority Act 707	Statutory law
7	Cybersecurity Act of Ghana 2020	Statutory law
8	Guidelines for the Use of CCTV^b^ in GHS Facilities	Guidelines
9	Health sector ICT^c^ policy and strategy	Policy

^a^GHS: Ghana Health Services.

^b^CCTV: closed-circuit television.

^c^ICT: information and communication technology.

**Table 6:** Legal documents from Indonesia.

No	Legal document	Type
1	Regulation of the Minister of Health of the Republic of Indonesia Number 269/2008 on Medical Record	Regulation
2	Undang-Undang Republik Indonesia Nomor 29 Tahun 2004 Tentang Praktik Kedokteran	Statutory law
3	Undang-Undang No. 36/2009 Pasal 103 ayat 1	Statutory law
4	Peraturan Menteri Kesehatan Republik Indonesia Nomor 55 Tahun 2013 Tentang Penyelenggaraan Pekerjaan Perekam Medis	Regulation
5	Undang-Undang Republik Indonesia No 36 Tahun 2014 Tentang Tenaga Kesehatan	Statutory law
6	Peraturan Pemerintah Republik Indonesia Nomor 46 Tahun 2014 Tentang Sistem Informasi Kesehatan	Regulation
7	UU 36 Tahun 2009 Tentang Kesehatan	Statutory law
8	Peraturan Menteri Kesehatan Republik Indonesia Nomor 36 Tahun 2012 Tentang Rahasia Kedokteran	Regulation
9	Undang-Undang Republik Indonesia Nomor 44 Tahun 2009 Tentang Rumah Sakit	Statutory law
10	Peraturan Menteri Kesehatan Republik Indonesia Nomor 82 Tahun 2013 Tentang Sistem Informasi Manajemen Rumah Sakit	Regulation
11	Peraturan Menteri Kesehatan Republik Indonesia Nomor 77 Tahun 2016 Tentang Sistem Klasifikasi Keamanan Dan Akses Arsip Dinamis Di Lingkungan Kementerian Kesehatan	Regulation

**Table 7:** Legal documents from the EU^a^.

No	Legal document	Type
1	Penal Code [41,69]	Case law
2	Directive 95/46/EC	Directive [70,71]
3	NIS^b^ Directive	Directive [72]
4	The directive on patients’ rights in cross-border health care (Directive 2011/24)	Directive [73]
5	Directive 2009/136/EC amending Directive 2002/58/EC (Privacy Directive)	Directive
6	Data Protection and Privacy in Electronic Communications—e-Privacy Directive (it replaces Directive 97/66/EC) [74]	Directive
7	Directive 99/93/EC	Directive [75]
8	The Patients’ Rights Directive (2011/24/EU) [73]	Directive
9	Recommendation CM/Rec(2019)2 of the Committee of Ministers to member states on the protection of health-related data [76]	Guidelines
10	GCP^c^	Guidelines [71]
11	Recommendation No. R (97) 5 of the Committee of Ministers to Member States on the Protection of Medical Data	Recommendation [77]
12	GDPR^d^ [16,78-83]	Regulation
13	EU regulation and compliance of national and transborder data flows	Regulation
14	Medical Device Regulation 2017/745 of EU [41]	Regulation
15	Regulation 2014/910 (the *eIDAS*^e^ *Regulation*) [78]	Regulation
16	A European standardization group for Security and Privacy of Medical Informatics (CEN TC 251/WG6^f^) [84,85]	Standard
17	GEHR^g^/CEN^h^ standards ENV^i^ 12265 and ENV 13606 [86,87]	Standard

^a^EU: European Union.

^b^NIS: Network and Information Security.

^c^GCP: Good Clinical Practice.

^d^GDPR: General Data Protection Regulation.

^e^eIDAS: electronic identification and trust services.

^f^CEN TC 251/WG6: Commission for European Normalization Technical Committee/Working Group 6.

^g^GEHR: Good European Health Record.

^h^CEN: European Committee for Standardization.

^i^ENV: Electronic Healthcare Record Communication for the exchange of electronic health records.

**Table 8:** International legal documents.

No	Legal document	Type
1	ISO^a^ 27001	Standard
2	IEC^b^ 80001-1:2010	Standard
3	The Universal Declaration of Human Rights	Statutory law

^a^ISO: International Organization for Standardization.

^b^IEC: International Electrotechnical Commission.

**Table 9:** Legal requirement used in the study.

No	Requirement	Count, n (%)	Reference
1	GDPR^a^	13 (21.67)	[16,78-82,88-94]
2	Directive 95/46/EC	10 (16.67)	[65,70,71,74,75,95-99]
3	Norwegian Personal Health Data Filing System Act	3 (5)	[16,100,101]
4	Act relating to Patients’ Rights	2 (3.33)	[16,101]
5	Act relating to the Processing of Personal Data	2 (3.33)	[16,101]
6	Directive 2011/24/EU on patients’ rights in cross-border health care	2 (3.33)	[73,90]
7	Health Care Personnel Act	2 (3.33)	[16,101]
8	Act relating to Public Supervision of the Health Service	1 (1.67)	[101]
9	Data protection and privacy in electronic communications—e-Privacy Directive	1 (1.67)	[75]
10	Directive 2002/58/EC	1 (1.67)	[65]
11	Directive 2009/136/EC	1 (1.67)	[74]
12	Directive 99/93/EC	1 (1.67)	[75]
13	EU regulation and compliance of national and transborder data flows	1 (1.67)	[89]
14	GEHR^b^/CEN^c^ standards ENV^d^ 12265 and ENV 13606	1 (1.67)	[102]
15	Good Clinical Practice	1 (1.67)	[71]
16	Health Research Act	1 (1.67)	[16]
17	IEC^e^ 80001-1:2010	1 (1.67)	[97]
18	ISO^f^ 27001	1 (1.67)	[89]
19	Medical Device Regulation 2017/745 of EU	1 (1.67)	[41]
20	Ministry Of Government Administration, Reform and Church affairs’ Requirements specification for PKI^g^ for the public sector	1 (1.67)	[65]
21	Penal Code	1 (1.67)	[41]
22	Recommendation CM/Rec(2019)2 of the Committee of Ministers to member States on the protection of health-related data	1 (1.67)	[76]
23	Recommendation No. R (97) 5 of the Committee of Ministers to Member States on the Protection of Medical Data	1 (1.67)	[77]
24	Regulation 2014/910 (the “eIDAS Regulation”)	1 (1.67)	[103]
25	Regulation of the Minister of Health of the Republic of Indonesia Number 269/2008 on Medical Record	1 (1.67)	[83]
26	Regulations relating to the Processing of Personal Data	1 (1.67)	[101]
27	The Ghana Health Services Patient’s Charter	1 (1.67)	[104]
28	The Ghana National Health Insurance Regulations of 2004	1 (1.67)	[104]
29	The National Identification Authority Act 707	1 (1.67)	[104]
30	The Republic of Ghana’s constitution	1 (1.67)	[104]
31	The Universal Declaration of Human Rights	1 (1.67)	[104]
32	UNDANG-UNDANG No.36/2009 and Pasal 103 ayat 1	1 (1.67)	[105]
33	Undang-undang republik, Indonesia nomor 29, Tahun 2004 tentang, Praktik kedokteran	1 (1.67)	[106]

^a^GDPR: General Data Protection Regulation.

^b^GEHR: Good European Health Record.

^c^CEN: European Committee for Standardization.

^d^ENV: Electronic Healthcare Record Communication for the exchange of electronic health records.

^e^IEC: International Electrotechnical Commission.

^f^ISO: International Organization for Standardization.

^f^PKI: public key infrastructure.

**Table 10:** Security requirement category distribution (n=173).

No	Security requirement category	Count, n (%)
1	Data processing	14 (8.1)
2	Data protection officer	14 (8.1)
3	Right of access	13 (7.5)
4	Security by design	13 (7.5)
5	Access control	12 (6.9)
6	Email processing	10 (5.8)
7	Logs	9 (5.2)
8	Password	7 (4.1)
9	Encryption	6 (3.5)
10	Health data storage	6 (3.5)
11	Mobile phone processing	4 (2.3)
12	Privacy by design	4 (2.3)
13	CIA^a^ measures	3 (1.7)
14	Data controller	3 (1.7)
15	Personal data	3 (1.7)
16	Third countries	3 (1.7)
17	Data protection	3 (1.7)
18	Backup	2 (1.2)
19	Documentation	2 (1.2)
20	Electronic signature	2 (1.2)
21	Establish security governance	2 (1.2)
22	Least privileges	2 (1.2)
23	Medical devices	2 (1.2)
24	Right to be informed	2 (1.2)
25	Risk management	2 (1.2)
26	Security governance	2 (1.2)
27	Third parties	2 (1.2)
28	Data breach	2 (1.2)
29	Use of ISO^b^ standards	2 (1.2)
30	Consent	1 (0.6)
31	Data aggregation	1 (0.6)
32	Incident reporting	1 (0.6)
33	Internal control	1 (0.6)
34	Data transfer to non-EU^c^ countries	1 (0.6)
35	Deletion of health data	1 (0.6)
36	Establish security policies	1 (0.6)
37	Health care data hosting	1 (0.6)
38	Identity	1 (0.6)
39	Internal and external threats	1 (0.6)
40	Mobile devices	1 (0.6)
41	Monitoring of NIS^d^ Directives	1 (0.6)
42	Patients from other member states	1 (0.6)
43	Physical security	1 (0.6)
44	Professional secrecy	1 (0.6)
45	Protection against security incidents	1 (0.6)
46	Providing information to patients from a member state	1 (0.6)
47	Risk assessment	1 (0.6)
48	Risk mitigation	1 (0.6)
49	Sanction	1 (0.6)
50	Technological security measures	1 (0.6)
51	Training and education	1 (0.6)

^a^CIA: confidentiality, integrity, and availability.

^b^ISO: International Organization for Standardization.

^c^EU: European Union.

^d^NIS: Network and Information Security.

**Table 11:** Privacy requirement category distribution (n=80).

No	Privacy requirement category	Count, n (%)
1	Consent	13 (16)
2	Disclosure of health data	12 (15)
3	Privacy by design	8 (10)
4	Right to privacy	8 (10)
5	Right of access	7 (9)
6	Data protection	6 (8)
7	Data processing	3 (4)
8	Personal data	3 (4)
9	Punitive measures of security and privacy violation	3 (4)
10	How to record health data	2 (3)
11	Privacy rights	2 (3)
12	Storage of health records	2 (3)
13	CIA^a^ measures	1 (1)
14	Data collection purpose	1 (1)
15	Deletion of health data	1 (1)
16	Electronic signatures	1 (1)
17	Mobile phone processing	1 (1)
18	Professional secrecy	1 (1)
19	Purpose of health care data processing	1 (1)
20	Right to be forgotten	1 (1)
21	Right to object	1 (1)
22	Termination of consent	1 (1)
23	Third parties	1 (1)

^a^CIA: confidentiality, integrity, and availability.

### Findings

The following sections present and describe a series of findings, including law by type, law by jurisdiction, requirement by type, requirement by responsibility level, and identified security and privacy requirements and their categorizations.

#### Law by Type

The types of laws identified in this work are presented in Multimedia Appendix 1 and Table 2. A total of 75 legal requirements were identified in this review. The most common types of laws that were used are policies (27/75, 36%), statutory law (16/75, 21%), regulations (13/75, 17%), directive (7/75, 9%), standards (4/75, 5%), and guidelines (4/75, 5%), but recommendation, code of conduct, charter, and case law accounted for the lowest proportion. It is worth noting that the 27 policies were all collected from information security policy documents of the health care facilities of the southeast region in Norway as their internal control measures of information security and privacy measures.

#### Law by Jurisdiction

The specific legal documents from Norway, Ghana, Indonesia, the EU level, and the international level are listed in Table 4, Table 5, Table 6, Table 7, and Table 8, respectively, and Norway has almost half (36/75, 48%) of the laws pertaining to information security and privacy, which were identified in this work and shown in Multimedia Appendix 2 and Table 4. This was followed by the EU (17/75, 23%). The southeast health region in Norway developed approximately 27 policies, which also accounted for the larger proportion of the laws in Norway than that in other countries, as shown in the bar chart of the law jurisdiction distribution in Multimedia Appendix 2.

#### Identified Legal Requirement

Of the 82 requirement sources, 36 (44%) were articles that considered at least one of the identified requirements, whereas the others were legal documents. In total, 75 unique legal documents were identified, and 33 legal documents were identified to have been considered in the papers as shown in Table 9.

Moreover, as shown in Table 9, among all the legal documents, the GDPR (13/60, 22%) is the most common regulation that was used in the articles that relied on legal requirements, followed by Directive 95/46/EC (10/60, 17%), which has already been repealed and replaced by the GDPR. Some acts from Norway, as well as directive from the EU, have also been referred to several times, such as the Norwegian Personal Health Data Filing System Act (3/60, 5%), Act relating to Patients’ Rights (2/60, 3%), Act relating to the Processing of Personal Data (2/60, 3%), Directive 2011/24/EU on patients’ rights in cross-border health care (2/60, 3%), and Health Care Personnel Act (2/60, 3%).

#### Security and Privacy Requirements

According to Multimedia Appendix 3, most legal requirements extracted are security requirements (173/253, 68.4%), whereas the rest are privacy requirements (80/253, 31.6%).

#### Requirements by Responsibility Level

The identified responsibility level of users includes management, end users, and all users. The management level has more security and privacy responsibility and stipulation than the end users. As shown in Multimedia Appendices 4 and 5, documents list the security and privacy requirements only for end users.

#### Security Category

The security requirements extracted from all the studies cover various aspects, such as data processing, data protection officer, right of access, security by design, access control, email processing, logs, and password, as shown in Table 10. In this study, security requirements relating to data processing (14/173, 8.1%), data protection officer (14/173, 8.1%), right of access (13/173, 7.5%), security by design (13/173, 7.5%), access control (12/173, 6.9%), email processing (10/173, 5.8%), logs (9/173, 5.2%), password (7/173, 4%), encryption (6/173, 3.5%), and health data storage (6/173, 3.5%) were identified to be commonly adopted in the legal requirements, as shown in Table 10.

#### Privacy Category

The privacy requirement categories that were realized in this work are shown in Table 11.

The areas that were mostly required by the legal instruments are consent (13/80, 16%), disclosure of health data (12/80, 15%), privacy by design (8/80, 10%), right to privacy (8/80, 10%), right of access (7/80, 9%), data protection (6/80, 8%), data processing (3/80, 4%) and punitive measures (3/80, 4%).

## Discussion

### Principal Findings

The main purpose of this study is to comprehensively identify, assess, and synthesize the appropriate legal requirements and security governance tools of information security to serve as a yardstick for modeling and analyzing health care security practices. A scoping review of these requirements was conducted to include various categories, as presented in Table 1. The most used categories identified in this study are listed in Table 12. For instance, among various types of laws that were identified in this study (Multimedia Appendix 1), the most used types of law are the policies, statutory law, regulations, and directives, as shown in Table 12.

**Table 12:** Summary of the most used categories.

No	Category	Most used
1	Type of law	Policy, statutory law, regulation, and directive
2	Jurisdiction	Norway and European Union
3	Requirement type	Security requirement
4	Responsibility level	Management
5	Security requirement category	Data processing, data protection officer, right of access, security by design, access control, email processing, logs, password, encryption, and health data storage
6	Privacy requirement category	Consent, disclosure of health data, privacy by design, right of access, and data protection

### Security Requirement Responsibility Level Distribution

As defined in Table 1, the responsibility level of the requirement is the level of user categories that take action to observe, enforce, implement, or comply with the security measure. Examples include management, end users, and all users. Management includes top-level staff, such as the chief executive officers (CEOs), directors, managers, and officers, who are responsible for implementing and observing health care security practices. All users include all employees, consultants, suppliers, and others with access to the health care system and with the responsibility to comply with security and privacy requirements. The end users’ level includes only those user categories that have access to the health care system with the purpose of accessing and performing specified tasks. Such users include nurses, doctors, pharmacies, record management, and patients’ EHRs for therapeutic reasons.

As shown in Multimedia Appendices 4 and 5, the management level was identified to be mostly responsible for information security and privacy requirements, followed by *all users* This implies that in most information security and privacy requirement categories such as access control, password management, consent, and incident reporting, as outlined in Tables 10 and Tables 11, the management level has more responsibility. The management user category includes the CEO, chief information officer, chief information security officer, all directors, and all managers responsible for formulating, designing, and implementing privacy and security policies for compliance [37]. The top-management user category, such as the CEO, chief information officer, and chief information security officer, is responsible for coming out with the information security governance requirement based on prevailing laws pertaining to information security. Directors and managers then ensure that the policies, guidelines, standards, and best practices are appropriately designed and implemented. They also need to create awareness and ensure that all personnel are adequately trained in these requirements. Essentially, impact assessments such as privacy and security are also conducted by the management. To ensure compliance, these policies need to be monitored and evaluated. Management, therefore, has a major proportion of responsibility because of all these broad activities being performed toward enhancing security.

In addition, the *all users* category consists of all employees such as the management level and end users including temporal workers and contractors who have the responsibility to enforce and comply with the requirements. The *all users* category of the level of responsibility involves requirements that need the attention of both management and end users. For instance, access control requires management to incorporate it into the development of systems. However, end users must also be responsible for their access control–related behaviors, including password management. The *end users* level includes those health care workers who are given access to a system based on their need to use that system for therapeutic purposes [61]. Examples include the end users of an EHR system. This group of users is mostly large in number but does not have an enormous number of responsibilities as compared with the management group, as shown in Multimedia Appendices 4 and 5.

### Requirement Types (Security and Privacy)

A total of 2 kinds of measures were extracted from the legal documents in this study, namely, security and privacy requirements. The legal documents contain at least one of the two kinds of measures: privacy, security, or both. Furthermore, >1 requirement was found in some of the sources of the legal documents, and this resulted in more legal requirements compared with the number of identified sources, as shown in Table 9. After the identification and extraction process, 173 security requirements and 80 privacy requirements were identified, as shown in Multimedia Appendix 3. The findings indicate that there are more security requirements than privacy requirements identified in this study. The main reason is that many policies in Norway describe security requirements, as shown in Multimedia Appendix 1 and Table 4. Most of these policies were developed to address security requirements such as email use, crypto policy, password policy, and access control logging, which resulted in the number of security requirements surpassing the number of privacy requirements.

### Law by Type

From Table 2, a total of 10 types of laws were identified in this study, including case law, charter, code of conduct, directives, guidelines, policies, and recommendations. Others include regulations, standards, and statutory law, of which the most used type of laws are policies (27/75, 36%), statutory law (16/75, 21%), regulations (13/75, 17%), directives (7/75, 9%), standards (4/75, 5%), and guidelines (4/75, 5%), as shown in Table 12. The standards that were identified are only from the EU and international levels with which Norway is bound to comply. In addition, none of the countries has standards as far as what we have collected. This could be due to the level of maturity of IT development in health care in each country. Finally, only a few documents were categorized into case law, charter, recommendation, and code of conduct.

One of the most influential legal documents that covers almost every general aspect, as mentioned is the GDPR, as shown in Table 9, to which data controllers, data processors, and data subjects need to comply. It is worth mentioning that pursuant to the GDPR, “a data controller is a legal person, public authority, agency or other body which, alone or jointly with others, determines the purposes and means of the processing of personal data,” whereas a data processor means a legal person, public authority, agency, or other body that processes personal data on behalf of the controller [107]. A data subject is any identified or identifiable person whose data are processed by the data processor. ISO 27001 provides a framework for managing security issues in health care including the measures covering information security policies, organization of information security, human resource security, asset management, media handling, access control, cryptography, physical and environmental security, operational security, communications security, system acquisition, development and maintenance, supplier relationships, and information security incident management through ISO 27799 [14]. Health care has extended needs in these areas, which is why ISO 27799 was developed for use in conjunction with ISO 27001. This provides room to address the security and privacy requirements that have not been fully covered in ISO 27001.

The widely used model, namely, the CIA triad, which is the balanced protection of CIA of data [108], is the foundation and basis of many laws and regulations including the GDPR, Recommendation CM/Rec (2019)2 of the Committee of Ministers to member states on the protection of health-related data, Directive 2009/136/EC amending Directive 2002/58/EC (Privacy Directive), Medical Device Regulation 2017/745 of EU, and Regulation 2014/910 (the *eIDAS Regulation*) at the EU level, as well as the Norwegian Personal Health Data Filing System Act, Act relating to the Processing of Personal Data, and Act relating to Patients’ Rights as shown in Table 9.

### Law by Country

The legal documents were identified from 3 countries: Norway, Ghana, and Indonesia. Norway has the most legal documents for this study at 47% (35/75), whereas Ghana and Indonesia provide only 12% (9/75) and 15% (11/75) of the documents, respectively. The main reason Norway has far more relevant legal documents than the other 2 countries is that Norway has many policies that describe specific details on security and privacy requirements. Furthermore, we also identified some legal documents from the EU (17/75, 23%) and some international laws (3/75, 4%). Most EU documents are directives and regulations that should be adopted by EU members, including Norway. Meanwhile, the international laws include 2 ISOs and 1 statutory law, which should be adopted by all countries.

### Security and Privacy Policies in Norway, Ghana, and Indonesia

The privacy requirements in this study focused on patients’ consent to the processing of their personal data and the processing and storage of medical records, as shown in Table 11. The requirements for processing personal information include that the data subjects must consent to the use of their data captured and collected in the first place [109]. Patients have the right to object to the processing of their personal health data (Norwegian Personal Health Data Filing System Act [110]) and are entitled to their information not to be disclosed to a third party without their consent [111]. The Health Research Act in Norway stipulates that more detailed requirements regarding consent must be informed, voluntary, express, and documented [112]. As for the processing of medical records, it is specifically stated in Indonesian laws that the medical data should be kept confidential by the management level to protect the patients and hospitals must protect archived physical records [106].

Security and privacy requirements in Norway, Ghana, and Indonesia all contain laws to protect the CIA of health care data. As shown in Multimedia Appendix 2, almost 46% (35/75) of the laws were developed by Norway, and most of the information security and privacy policies were developed by Norwegian health care facilities to meet the CIA requirements of health care data and information, as compared with Indonesia and Ghana. The variance could arise from various reasons, including advancement in the application of ICT in health care between European and African countries [113,114], and culturally related factors among the 3 countries. Norway is one of the countries in Europe that might have been more advanced in the use of ICT in health care than Ghana and Indonesia and have therefore adopted more legal requirements than Ghana and Indonesia. In addition, Norway is affiliated with the EU through the European Economic Area and is therefore bound to adopt the legal requirements, such as the GDPR and Network and Information Security Directive. In addition, EU countries, including Norway, are concerned with privacy [114]. This may have been one of the reasons for the adoption of more legal requirements to comprehensively enhance privacy and security measures.

### Framework

**Figure 2:**
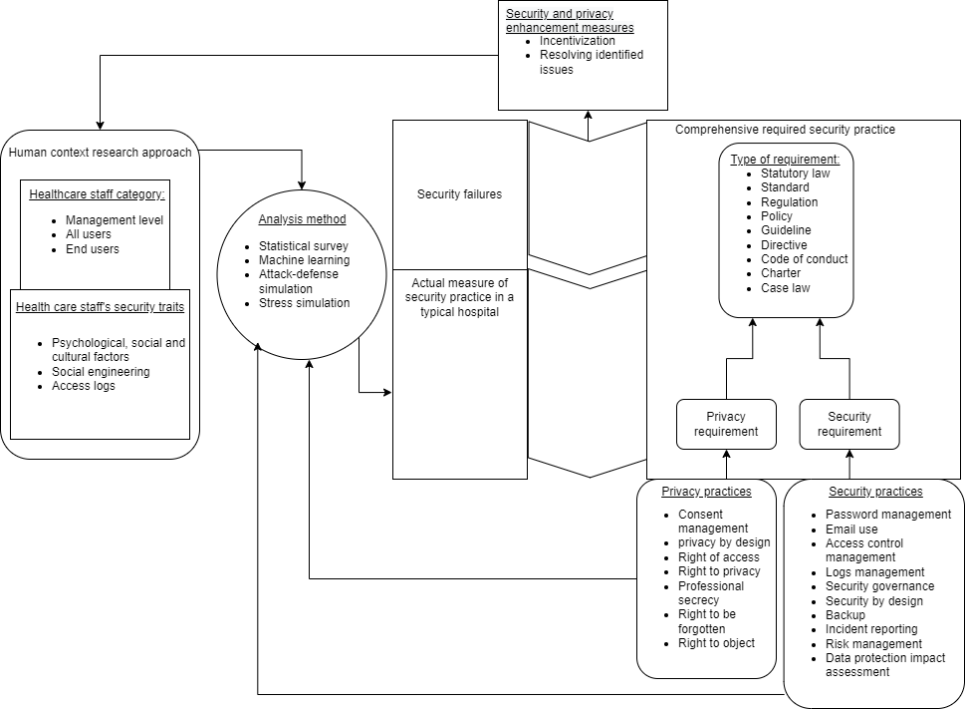
Legal requirement framework.

**Figure 3:**
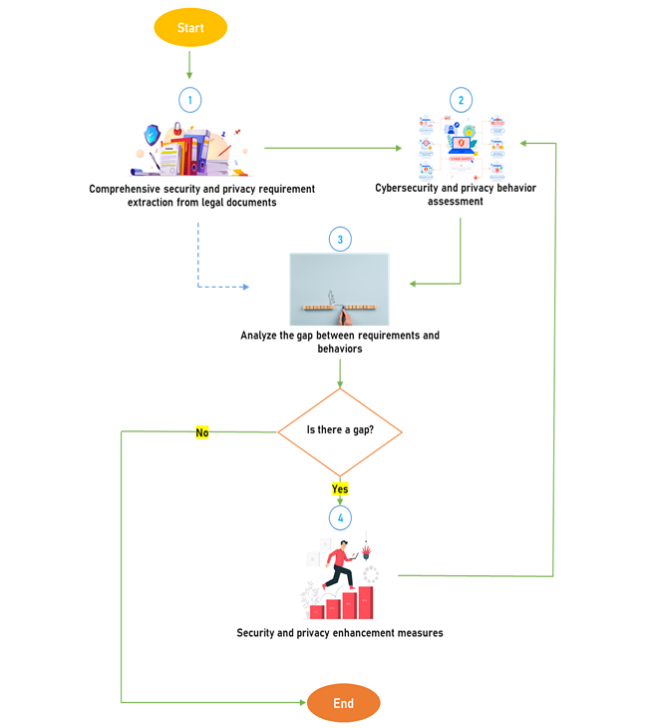
Measurement flowchart.

On the basis of our findings on security requirements, we present a framework in this section to provide directions for future imperial research in health care security practices. The framework consists of comprehensive security practices (drawn from the security requirements) and categories of health care staff in health care information security practices. It also includes analysis methods, the actual measure of security practices in a typical hospital, a gap or security failures, and an incentivization module, as shown in Figures 2 and 3 and as described as follows:

- Comprehensive security requirements: these include both privacy and security requirements that have been identified in the legal and security governance requirements in this study, as shown in Tables 10 and 11. These requirements are to be observed by all categories of health care workers. These requirements serve as the benchmark to be complied with by all categories of health care staff.
- Categories of users: these include management, all users, and the end users of a typical hospital. These categories of users must observe the required security practices at their respective levels, as shown in Figure 2.
- Analysis methods: in assessing health care security practices, various methods must be identified and used, as shown in Figure 2. These include a hybrid survey consisting of both qualitative and quantitative approaches [6,16-115]. Attack-defense simulation is when the investigator acts as the adversary to gain access to health care resources by using various techniques, including social engineering, brute-force attack, and SQL injection, depending on the goal of the attacker. Data analysis with machine learning can also be adopted to analyze logs of health care staff to determine abnormal access and maliciousness. The analysis method obtains inputs from the comprehensive required security and privacy practices fused with the various levels of health care staff user categories.
- In addition, health care staff have various characteristics that can be traced in the psychological-social and cultural contexts, social engineering, and access logs [16].
- These qualities also serve as input to the study approach.
- The actual measure of security practices was then determined from the assessment and compared with the required security and privacy practices.
- Security failures are gaps or deltas in the security practices that are determined if, after assessment, the hospital is not able to fully comply with all the identified requirements.
- Security and privacy enhancement measures: security failures can be improved with security and privacy enhancement measures, such as incentive measures and improving on factors that influence security failures. For instance, health care staff can be treated with various incentivization measures to improve their security-conscious care behavior. The assessment can then be conducted to determine the effectiveness of the treatment.

Information security and privacy requirements change based on or assessed threats, thus requiring changes in various laws. Therefore, the framework is such that the study can always be repetitive, as shown in Figure 2, to assess and identify related security and privacy gaps among health care workers in their application of ICT in health care. In Figure 2, the framework implementation is simplified, and security requirements are identified for security and privacy behavior assessment. The findings were compared with the required security behavior. Identified gaps can always be improved through cybersecurity and privacy incentives.

### Conclusions

Amidst various information security solutions, data breaches continue to increase, especially in the area of the health care staff information security practice. This has attracted research interest in modeling and assessing health care staff’s information security practices toward improving their security-conscious care behavior.

However, there is no holistic benchmark that serves as a yardstick in assessing health care information security practices comprehensively. To this end, we systematically reviewed information security requirements in health care in the context of legal requirements and information security governance tools for comprehensive security and privacy requirements in health care in Norway, Indonesia, and Ghana. Approximately 173 security requirements covering data processing, right of access, security by design, access control, email processing, logging, password, encryption, health care data storage, data processing officer, and so on were identified, as shown in Table 10.

In addition, approximately 80 privacy requirement categories were identified and included consent, disclosure of health data, privacy by design, right to privacy, right of access, data protection, data processing, personal data, and punitive. measures, as shown in Table 11. On the basis of these findings, a framework for modeling, analyzing, and developing effective security countermeasures, including incentivization measures, was developed, as shown in Figures 2 and 3. Following this framework, research results of health care security practices would be more reliable and effective than relying on incomprehensive security requirements. However, we observed some limitations that should be considered in future studies. For instance, there may be more standards in information security, but we focused on health care–related information security standards from the scientific papers that we searched for based on the scope we set. Therefore, it may not be an exhaustive list of information security standards. Although we have identified the requirements and practices, in this framework, our work has not taken measures to narrow down the gap between requirements and practices by way of a real implementation. This is another limitation, and will be the next step in future work.

Having postulated this, the framework must be implemented to assess its effectiveness for general use. This framework will serve as a guideline for assessing security practices in health care.

## Appendix

Multimedia Appendix 1 Law type distribution.

Multimedia Appendix 2 Law jurisdiction distribution. EU: European Union.

Multimedia Appendix 3 Requirement type distribution.

Multimedia Appendix 4 Security requirement responsibility level.

Multimedia Appendix 5 Requirement type distribution.

## Abbreviations

CEO: Chief Executive Officer
CIA: confidentiality, integrity, and availability
EHR: electronic health record
EISP: enterprise or organizational information security policy
EU: European Union
GDPR: General Data Protection Regulation
ICT: information and communication technology
ISO: International Organization for Standardization
ISSP: issue-specific security policy
IT: information technology
PRISMA: Preferred Reporting Items for Systematic Reviews and Meta-Analyses
