# Supplementary figures and images for: Assessing the Legal Aspects of Information Security Requirements for Health Care in 3 Countries: Scoping Review and Framework Development
Source: JMIR Hum Factors. 2022 May 25;9(2):e30050. doi: 10.2196/30050 (PMC9178444; doi:10.2196/30050)

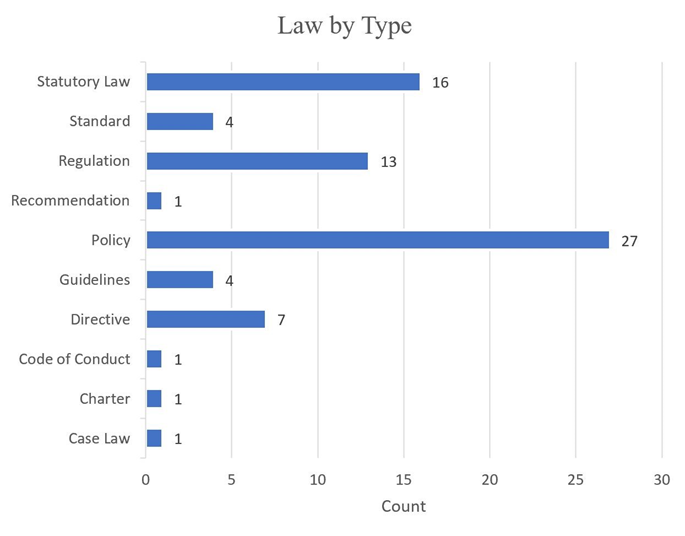

Supplement: Multimedia Appendix 1 [file humanfactors_v9i2e30050_app1.png]

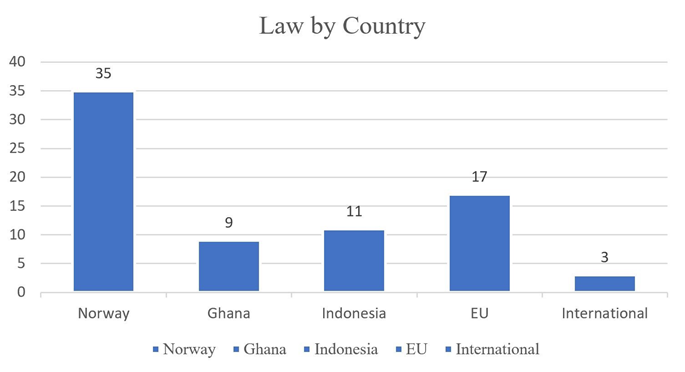

Supplement: Multimedia Appendix 2 [file humanfactors_v9i2e30050_app2.png]

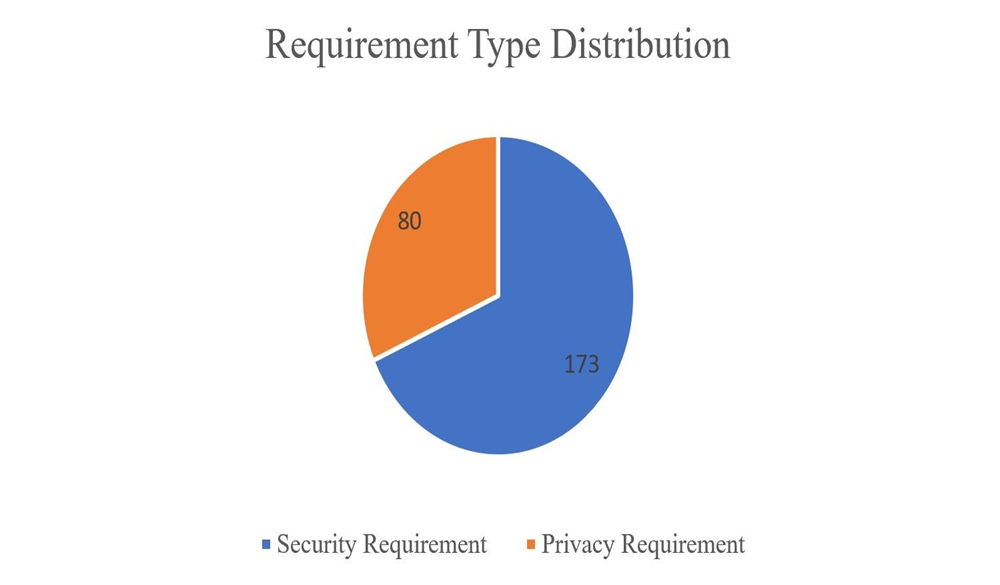

Supplement: Multimedia Appendix 3 [file humanfactors_v9i2e30050_app3.png]

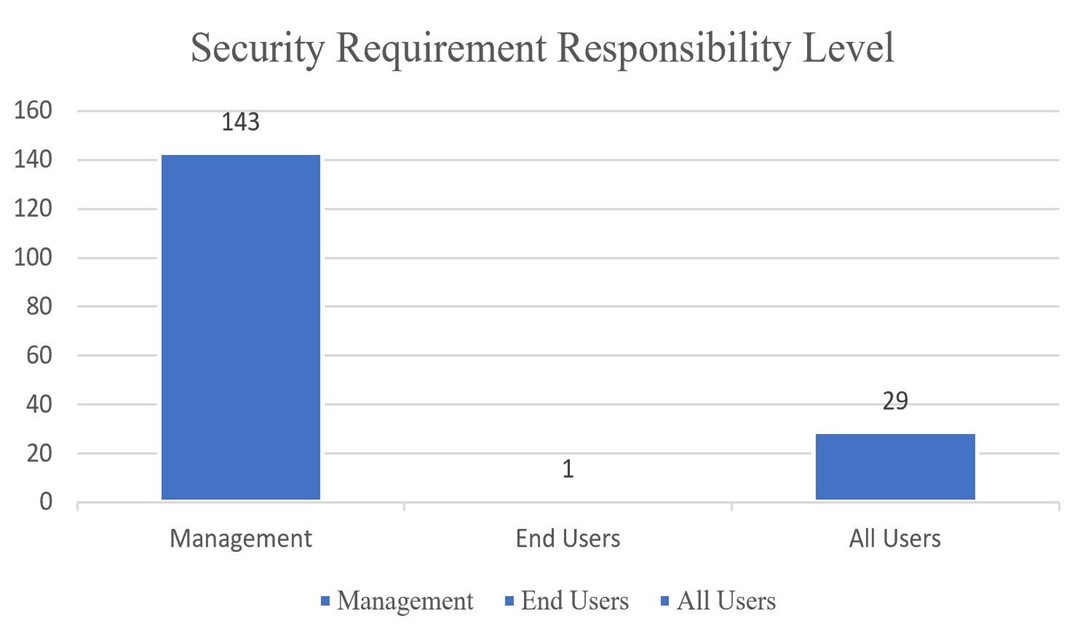

Supplement: Multimedia Appendix 4 [file humanfactors_v9i2e30050_app4.png]

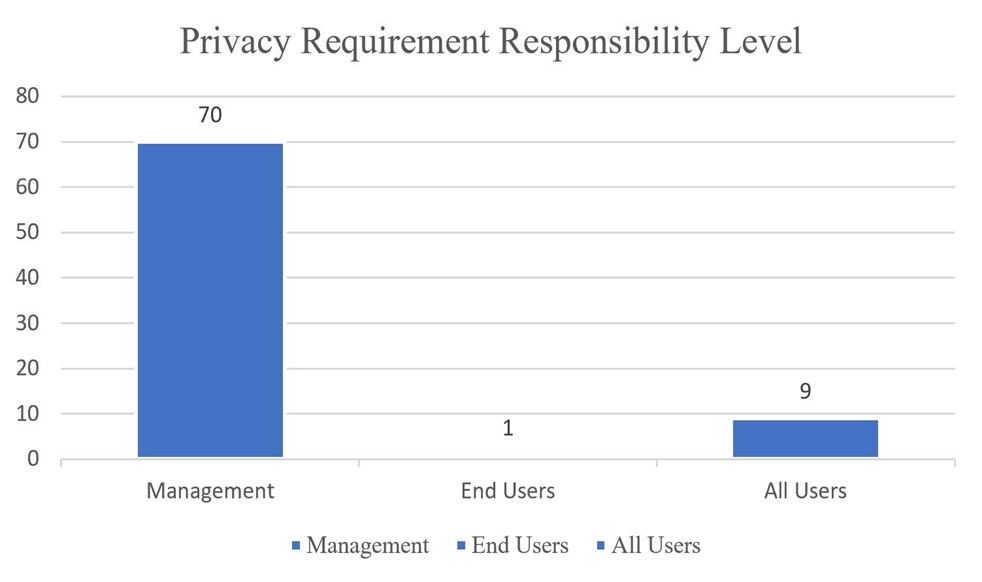

Supplement: Multimedia Appendix 5 [file humanfactors_v9i2e30050_app5.png]
